# A multiscale 3D finite element analysis of fluid/solute transport in mechanically loaded bone

**DOI:** 10.1038/boneres.2016.32

**Published:** 2016-09-27

**Authors:** Lixia Fan, Shaopeng Pei, X Lucas Lu, Liyun Wang

**Affiliations:** 1 Department of Mechanical Engineering, University of Delaware, Newark, DE, USA; 2 School of Mechanical Engineering, Nanjing University of Science and Technology, Nanjing, China

## Abstract

The transport of fluid, nutrients, and signaling molecules in the bone lacunar–canalicular system (LCS) is critical for osteocyte survival and function. We have applied the fluorescence recovery after photobleaching (FRAP) approach to quantify load-induced fluid and solute transport in the LCS *in situ*, but the measurements were limited to cortical regions 30–50 μm underneath the periosteum due to the constrains of laser penetration. With this work, we aimed to expand our understanding of load-induced fluid and solute transport in both trabecular and cortical bone using a multiscaled image-based finite element analysis (FEA) approach. An intact murine tibia was first re-constructed from microCT images into a three-dimensional (3D) linear elastic FEA model, and the matrix deformations at various locations were calculated under axial loading. A segment of the above 3D model was then imported to the biphasic poroelasticity analysis platform (FEBio) to predict load-induced fluid pressure fields, and interstitial solute/fluid flows through LCS in both cortical and trabecular regions. Further, secondary flow effects such as the shear stress and/or drag force acting on osteocytes, the presumed mechano-sensors in bone, were derived using the previously developed ultrastructural model of Brinkman flow in the canaliculi. The material properties assumed in the FEA models were validated against previously obtained strain and FRAP transport data measured on the cortical cortex. Our results demonstrated the feasibility of this computational approach in estimating the fluid flux in the LCS and the cellular stimulation forces (shear and drag forces) for osteocytes in any cortical and trabecular bone locations, allowing further studies of how the activation of osteocytes correlates with *in vivo* functional bone formation. The study provides a promising platform to reveal potential cellular mechanisms underlying the anabolic power of exercises and physical activities in treating patients with skeletal deficiencies.

## Introduction

It is well known that bone tissue is capable of adapting its mass and structure in response to mechanical cues.^1^ Although the cellular and molecular mechanisms of how the mechanical environment affects bone tissue are still not well understood, various mechanical parameters including (but not limited to) matrix deformations (strains), interstitial fluid pressure, and fluid flow-induced shear and drag forces have been found to impact bone’s responses to mechanical loading at cellular and tissue levels.^2^ Quantification of the strains associated with physiological mechanical stimuli in bone has been performed at both tissue and cellular levels.^3–5^ Using strain gages, the tissue-level strains were found to vary from ~600 μƐ during the light activity of walking to ~2 000 μƐ during vigorous activities such as running and jumping.^3,4^ Strains at the cellular levels have been mapped recently using finite element analysis (FEA) or imaging correlation techniques, and inhomogeneous strains (0.8%–3%) were recorded near the lacunar pores.^5^ Whether these matrix strains directly excite osteocytes, the presumed mechanical sensors in bone remain debatable. Previous experiments have shown that bone cells were more sensitive to loading-induced fluid flow than matrix strains.^6,7^ Recent studies strongly suggest that osteocytes, due to their large number and strategic positioning in the bone matrix, have very important roles in bone adaptation and metabolism.^2,8–10^ These multi-functioning cells form an extensive and well-connected network that optimizes them for detecting external mechanical stimuli, for example, fluid flow in the canaliculi driven by load-induced matrix deformations. In response, osteocytes release various soluble bioactive factors, which modulate the functions of other bone cells and trigger biological processes such as osteoclastic resorption during overuse and disuse as well as load-induced osteoblastic bone formation.^11–13^ The microscopic lacunar–canalicular system (LCS) that houses the osteocytes within the mineralized matrix is one key feature that enables the osteocytes to perform these important functions.^2,9,10^ Not only does the LCS provide the critical “life line” for nutrient supply and “network” for cell signaling^14–16^ but it also possesses the structural components (for example, tethering fibers within the fluid annulus) to amplify the loading signals and convert the overall loading to cellular stimulation forces such as shear stress and/or drag force acting on the osteocytes.^17,18^

We and others have built mathematical models to predict the magnitudes of load-induced fluid flow and solute transport in the LCS^19–26^ based on the groundbreaking paper by Weinbaum *et al.* ^8^ A mathematical model was developed to investigate the net solute (for example, nutrients and tracers) transport in the discrete LCS channels during cyclic loading of bone, and solute mixing within the extracellular space in lacunae was found to be responsible for the net solute transport.^25^ To guide the experimental investigation of solute convections using the fluorescence recovery after photobleaching (FRAP) approach that was developed initially for diffusion studies,^16^ Zhou *et al.* ^26^ developed a one-dimensional, three-compartment model to simulate load-induced solute transport in the LCS during FRAP experiments. This modeling work enabled the use of experimental FRAP measurements at the lacunar level to predict fluid flow in the canalicular level in a later study.^27^ We further expanded the three-compartment model by considering the solute–matrix interaction as exogenous tracer probes moved through the osteocytic pericellular matrix,^28^ allowing us for the first time to quantify the average fiber spacing in bones from young and aged mice with normal or altered pericellular matrix conditions.^29^ However, due to limited laser penetration into mineralized bone tissue, FRAP measurements have been limited only to the cortical bone 30–50 μm underneath the periosteum of the tibial cortex.^16,27–29^ Currently, transport measurements on deeper cortex and trabecular bone areas are lacking. These locations are of biological significance because they typically undergo changes under mechanical loading or disuse conditions.^30,31^

FEA is a powerful numerical tool of analyzing stress–strain fields in objects of irregular shapes and has been extensively used in bone research. A multilevel FEA model of a human femur cortex was developed to predict local matrix deformations at the osteocyte level during normal gait.^32^ To predict interstitial fluid flow in bone, a two-dimensional FEA poroelasticity model was developed to investigate the load-induced fluid flow in cortical bone.^21^ In another study, solute/fluid flow fields were predicted in an FEA model of a rat tibia under four-point bending.^33^ All of these models have focused on cortical bone sites. In this study, we aimed to develop a multiscale approach combining FEA and ultrastructural modeling. Our objective was to test the feasibility of the multiscale approach to predict functional outcomes resulted from mechanical loading. These outcomes include macroscopic (whole-bone level) and microscopic (LCS level) distribution fields of matrix strains, interstitial fluid pressure, as well as stimulation (fluid shearing and drag forces) at the cellular level. The rich pool of experimental data that we have obtained using FRAP on cortical bone^16,27,28,34^ was used to validate the material properties assumed in the multiscale model. This greatly improved the fidelity of our model predictions on deeper cortex and trabecular bone regions, which are inaccessible with current FRAP techniques.

## Materials and methods

### Whole-bone model

**Figure 1:**
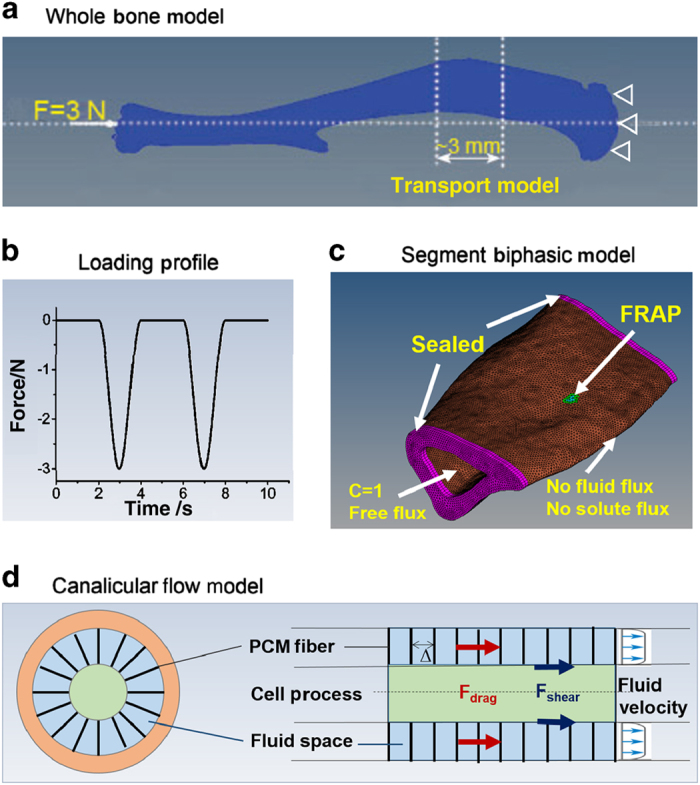
A multiscale model for loaded bone. (**a**) The whole-bone FEA model of a murine tibia with 5 112 690 tetra elements and its loading and boundary conditions. A 3 mm segment (20%–40% distal of the proximal end) was used for detailed analysis of strain, pore pressure, and fluid/solute fluxes. (**b**) The loading profile of cyclic compressive load 3N at 0.5 Hz followed by a 2 s resting periods. (**c**) The segment biphasic transport model was consisted of ~700 000 tetra elements with its fluid/solute boundary conditions. The site corresponding to the FRAP experiments is shown here. (**d**) Ultrastructural Brinkman flow model at single canaliculus (adapted from Weinbaum *et al.* ^8^) was used to predict fluid shear and drag force acting on osteocytes. The three levels of models are physically connected: the displacement outputs from the whole-bone model were used as boundary conditions in the segment transport model that provided fluid/solute flow at the tissue level, which were then converted to the canalicular level scaled with the LCS porosity.

An intact mouse tibia from an adult C57BL/6J male mouse was imaged by a Scanco μCT35 scanner (Scanco USA, Inc., Wayne, PA, USA) using a standard protocol (55 keV, 145 μA, 200 ms integration time, 3 600 projections, and 20 μm voxel size). The raw image slices (998 slices) were imported in the DICOM format into ScanIP (Simpleware, Chantilly, VA, USA), with which the entire tibia, including the cortical and trabecular bone, was thresholded and meshed with 5 112 690 tetra elements (Figure 1a). In Hypermesh (Altair/HyperWorks; http://www.altairhyperworks.com/), fixed displacement constraints were imposed at the elements of the proximal tibial plateau. Similar to our experimental setup,^27^ a 3 N compressive load was applied to the distal end of the tibia (Figure 1a). Assuming bone elements to be an elastic material with 20 GPa Young’s modulus and 0.33 Poisson’s ratio,^8^ the strain field was obtained using OptiStruct, a FEA linear solver in the HyperWork software package. The average strain of a 1×3 mm area on the medial–anterior surface that was 20%–40% distal from the tibial proximal end was compared with the strain measurement of a similar area in our previous studies.^27,35^ Good agreement between the comparisons would validate the material properties and boundary conditions assigned to the whole-bone FEA model.

### Segment biphasic model

For the analysis of fluid and solute transport in LCS, a 3 mm segment of the tibial metaphysis consisting of ~700 000 tetra elements was cut from the whole-bone FE model (20%–40% distal of the proximal end, Figure 1c). The elements were modeled as a biphasic porous elastic material in the FEBio (version 2.0; http://febio.org/), which is an open source finite element platform containing unique features that are suitable for modeling of mechano-chemical phenomena in biological tissues.^36^ The governing equations include the mass balance of the solid-solute mixture, the mass balance of solute, and the conservation of momentum as follows:

(1)div(vs→+w→)=0

(2)∂(φwk˜c˜)∂t+div(j→+φwk˜c˜vs→)=0

(3)grad(p˜+Rθ∅k˘c˜)+divσe→=0

where $\overset{\to }{v^{s}}$ is the velocity vector of the solid matrix, $\vec{w}$ is the volumetric flux of the solvent relative to the solid, $\varphi^{w}$ is the volume fraction of solvent in the mixture, $\overset{˘}{\kappa }$ is the effective solubility, $\tilde{c}$ is the effective solute concentration in the mixture, $\tilde{p}$ is the effective fluid pressure, *R* is the universal gas constant, *θ* is the absolute temperature, ∅ is the osmotic coefficient (a non-dimensional function of solute concentration and solid strain), and $\overset{\to }{\sigma^{e}}$ is the stress tensor arising from the strain in the porous solid matrix. The constitutive relations for the solid phase, water, and solutes can be referred to the theory manual of FEBio (www.febio.org) or the biphasic theory for cartilage.^37^

The model elements were defined as an isotropic porous linear elastic material (20 GPa modulus, 0.33 Poisson’s ratio) saturated with interstitial fluid (viscosity=0.001 Pa·s).^8^ As reviewed previously,^9^ there are three levels of porosity in the bone tissue: the large vascular pore (order 10 μm), the lacunar–canalicular pores (order 1–0.1 μm), and inter-collagen hydroxyapatite pores (order 1–10 nm). These intertwined pores make direct measurements of bone permeability quite challenging. The reported permeability varied from 10^−12^ to 10^−23^ m^2^, depending on the levels of pores probed.^9,38,39^ Because the murine cortex is relatively thin with pores being predominantly smaller LCS ones, the permeability at the tissue (element) level was chosen to be in the lower range of the reported values associated with the smaller pores. In the model, the permeability was varied parametrically over three orders of magnitude (2.8×10^−20^, 2.8×10^−21^, or 2.8×10^−22^ m^2^). The permeability was assumed to be isotropic and identical at both trabecular and cortical bones. Solute diffusivity was assumed to be isotropic for small strain cases as in loaded tibia.^27^

### Measurements of LCS porosity in the murine cortical bone

One key parameter for the above poroelastic FEBio modeling is the volume fraction of the LCS pores. Large variations of LCS porosity (1%–22%) were reported in literature using either two-dimensional or 3D imaging techniques.^39^ We thus measured the volume fractions of lacuna and canaliculi in the murine cortical bone using *in situ* confocal microscopy and a protocol modified from a previous study.^40^ In brief, femoral mid-shafts were dissected and sectioned into 0.3 mm-thick segments, fixed in 10% formaldehyde for 24 h, polished to 0.05–0.10 mm thickness, dehydrated in ascending grades of ethanol, stained in sodium fluorescein solution for 4 h, and mounted on an imaging chamber with a bottom cover glass. High-resolution 3D stacks (122 slices) of a field of 512×256 pixels (pixel 0.199 μm; *z*-step=0.2 μm), which contained several lacunae and numerous canaliculi, were captured using a 60× oil objective and a Zeiss LSM 510 confocal microscope (Carl Zeiss Microscopy, LLC, Peabody, MA, USA). The raw images were then imported to the Volocity software package (PerkinElmer, Tempe, AZ, USA), where an adaptive threshold and a size-dependent segmentation scheme were applied sequentially to separate the larger lacunae from the smaller canaliculi. The surface area of the lacunae and the total volume of the canaliculi was then obtained. Unlike previous measurements, we quantified the volume fraction of the LCS pores by subtracting the cell body volume. Our previous transmission electron microscopy studies showed that within the lacunae there was a 0.49±0.15 μm gap between the lacunar matrix wall and the cell body, and that the pericellular fluid space occupied 79% of the total canalicular cross-sectional area.^15^ These measures were used to calculate the volume fractions of the lacunar pores and the canalicular pores, respectively. The lacunar pore volume was the product of the total lacunar surface area and the lacunar gap. The total canalicular pore volume was a fraction (79%) of the total measured canalicular volume. Our result (shown later in the Results section) demonstrated a total lacunar and canalicular porosity (15.4%) in mouse, where the majority was contributed by canaliculi (87.5%). This value was adopted for all the simulations shown below. Please note that this value may represent an overestimation of the LCS porosity due to potential artifacts such as the partial volume effect and axial distortion associated with light microscopy.^41^

### Simulation of FRAP experiments: validation of the segment model

As part of validation of the biphasic transport model, we simulated FRAP experiments as performed in our previous experiments^27^ because they provided the most relevant experimental data in literature. One element (20 μm^3^), which was ~30 μm below the anterior–medial periosteum and similar to the dimensions of a single lacuna, was chosen to be the photobleached lacuna (the center of the highlighted green area, Figure 1c). The immediate post-photobleaching tracer concentration of the photobleached element and those within the 90^o^ laser cone below and above the photobleached element were set to be 0.2 due to the effects of photobleaching, whereas a concentration of 1.0 was assigned to the rest of elements in the model. Due to the relatively small hydraulic conductance and solute permeability of the mineralized bone tissue, as a first estimation, impermeable boundary conditions (no fluid and solute flux) were assumed for the periosteal surface and the bone cross-sections at the two ends of the model (Figure 1c).^26^ The marrow cavity was assumed to have a constant pressure and tracer concentration due to the presence of interstitial osmotic/hydraulic pressures and the tracer-rich vasculature, respectively. A free draining solute/fluid flux was assumed on the endosteal surface (Figure 1c).^25^ Transport of solutes with diffusivity varying from 27 to 100 μm^2^·s^−1^ was simulated as below.

Two FRAP experiments under non-loaded and loaded conditions^27^ were simulated using the transport model. For the non-loaded condition, the model was run for a total duration of 36 s with a time step of 0.2 s. The time course of solute concentration in the photobleached element was obtained from the simulation results. This time course was predicted to be an approximately exponential function of time.^16,27^ A transport rate *K*_diff_, was defined as the slope of the curve of ln[(*C−C*_0_)/(*C*_b_ *−C*_0_)] vs time, where *C* was the concentration at time *t*, *C*_0_ (=1) the concentration before photobleaching, and *C*_b_ (=0.2) the concentration immediately after photobleaching, *K*_diff_ measured the speed of the tracer recovery, which was the reciprocal of the time constant for the exponential recovery.^16^ Based on the experimental result of *K*_diff_ (=0.017 s^−1^) for sodium fluorescein,^27^ a best-fit diffusion coefficient was determined and compared with that obtained using the mathematical model developed previously.^16^ For the loaded condition, we first obtained the dynamic displacements on the two transverse surfaces that comprise the distal and proximal boundaries of the transport model (indicated by the dashed lines in Figure 1a and the purple surfaces in Figure 1c). These data were obtained from the whole-bone model simulation under the 3N peak load (Figure 1b) with a 0.5 Hz sinusoidal waveform followed by 2 s resting periods (total 4 s for a cycle). Up to eight cycles of loading were simulated. To capture the enhanced convective transport into photobleached lacuna through both loading and unloading phases of the loading cycle,^26^ the local solute concentration was superimposed with the two phases in sequence, as mixing in larger lacuna helped the entrapment of the tracer locally.^25^ A total of eight loading cycles (32 s) were simulated, from which the transport enhancement (*K*_load_ */K*_diff_) was obtained for sodium fluorescein and compared with the experimental measurements.^27^ A good agreement between the modeling results and the experimental data would justify the use of material properties assumed in the FEBio model.

### Outputs from the segment model

Once validation of the transport model was confirmed, the spatiotemporal distributions of pore pressure and fluid fluxes at the tissue level were obtained from the FEA model under cyclical mechanical loading. Several locations in the mid-transverse plane were chosen to show the results at both cortical and trabecular sites.

### Outputs from the ultrastructural canalicular flow model

To accomplish the goal of predicting the load-induced mechanical stimulation on osteocytes located on the entire bone, the outputs from the above segmental transport model, which reflected the tissue-level measures, were converted onto the cellular level. The tissue-level fluid fluxes were scaled to the canalicular level by a factor of 6.5 based on the average LCS porosity (15.4%), that is, the canalicular-level fluid flux would be 6.5 times of that at the tissue level predicted by the FEBio model. Using the Brinkman fluid flow model for a single canaliculus developed by Weinbaum *et al.*,^8^ we were able to predict the forces acting on the osteocytes including the shearing force and fluid drag force.^17^ The essential component of the Weinbaum model was the presence of fibers/tethers spanning the annular fluid space between the membrane of the cell process and the mineralized wall (Figure 1e). Recent studies have measured the radii of the canaliculi (160 nm) and osteocyte process (76 nm) in adult mouse bone,^18^ and a fiber spacing (center to center 14.3 nm).^29,42^ The formula of the canalicular flow profile, the shear stress, the shearing force, and drag force per unit length were given in the appendix of reference.^43^ As the tissue-level flow was found to be sensitive to tissue permeability, the sensitivity of the canalicular flow velocity was also tested by varying the permeability over three orders of magnitude (2.8×10^−20^, 2.8×10^−21^, and 2.8×10^−22^ m^2^).

## Results

### Model validations

#### Validating the whole-bone FEA model

**Figure 2:**
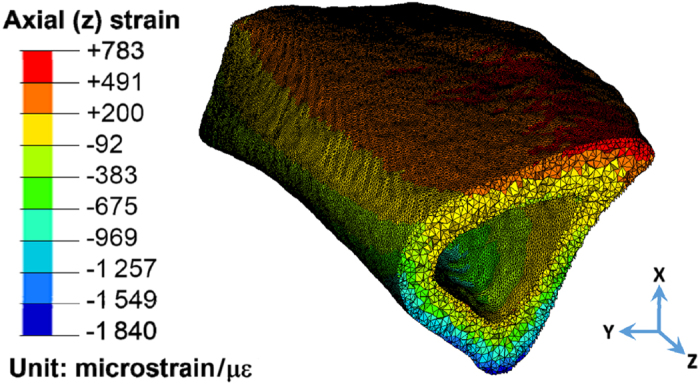
Axial strains from the whole-bone FE model. Only the portion corresponding to the segment model is shown. Due to the combination of compression and bending, the anterior–medial surface (FRAP site) was under tension.

The intact tibia bone was deformed by a combined mode of compression and bending under the 3 N compressive load applied at the ends. A tensile strain of ~450 μƐ was predicted on the relatively flat anteromedial tibial surface around the FRAP site (30% distal from the proximal end), and compressive strains were mostly found on the posterior cortex (Figure 2). In this linear FEA model, the strain values were proportional to the assumed Young’s modulus. Our predicted tensile strain of 450 μƐ at the region of interest (FRAP site) matched well with the experimentally measured data of ~400 μƐ.^27^ Thus, we concluded that the assumed material properties of 20 GPa Young’s modulus and 0.33 Poisson’s ratio were justified for the subsequent transport modeling.

#### Measuring the LCS porosity (an input to the segment model)

**Figure 3:**
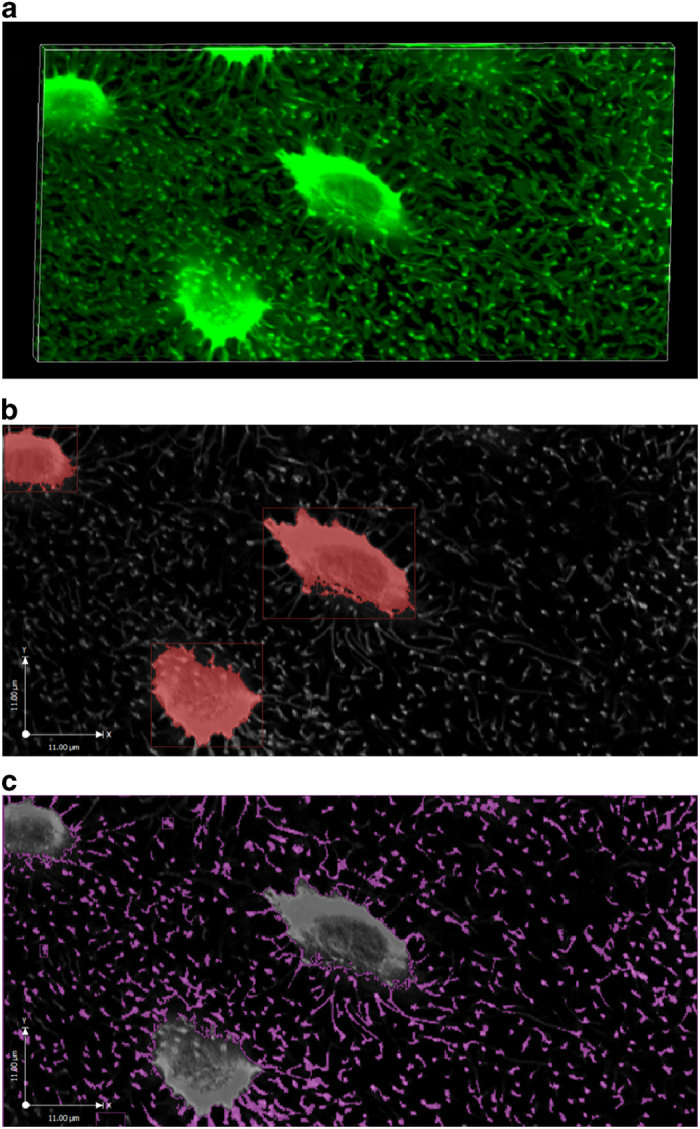
Confocal imaging of the murine cortical LCS. A stack of LCS images (**a**) was acquired. Individual lacuna (**b**) and canaliculi (**c**) were segmented and pore volumes measured.

Extensive LCS pores were labeled with high-intensity green fluorescence in the high-resolution 3D stacks of confocal images (Figure 3a), and the images were thresholded and segmented into lacunae and canaliculi categories (based on size criteria) for calculation of the porosity (Figure 2b and c). The volume fractions of the lacunae and canaliculi were found to be 1.9% and 13.5%, respectively. The total LCS porosity (15.4%) was then used as an input to the segment model. This porosity was also used to scale the tissue-level fluid flow predicted by the segment model to that at the canalicular level in the ultrastructural model.

#### Validating the segment biphasic model

**Figure 4:**
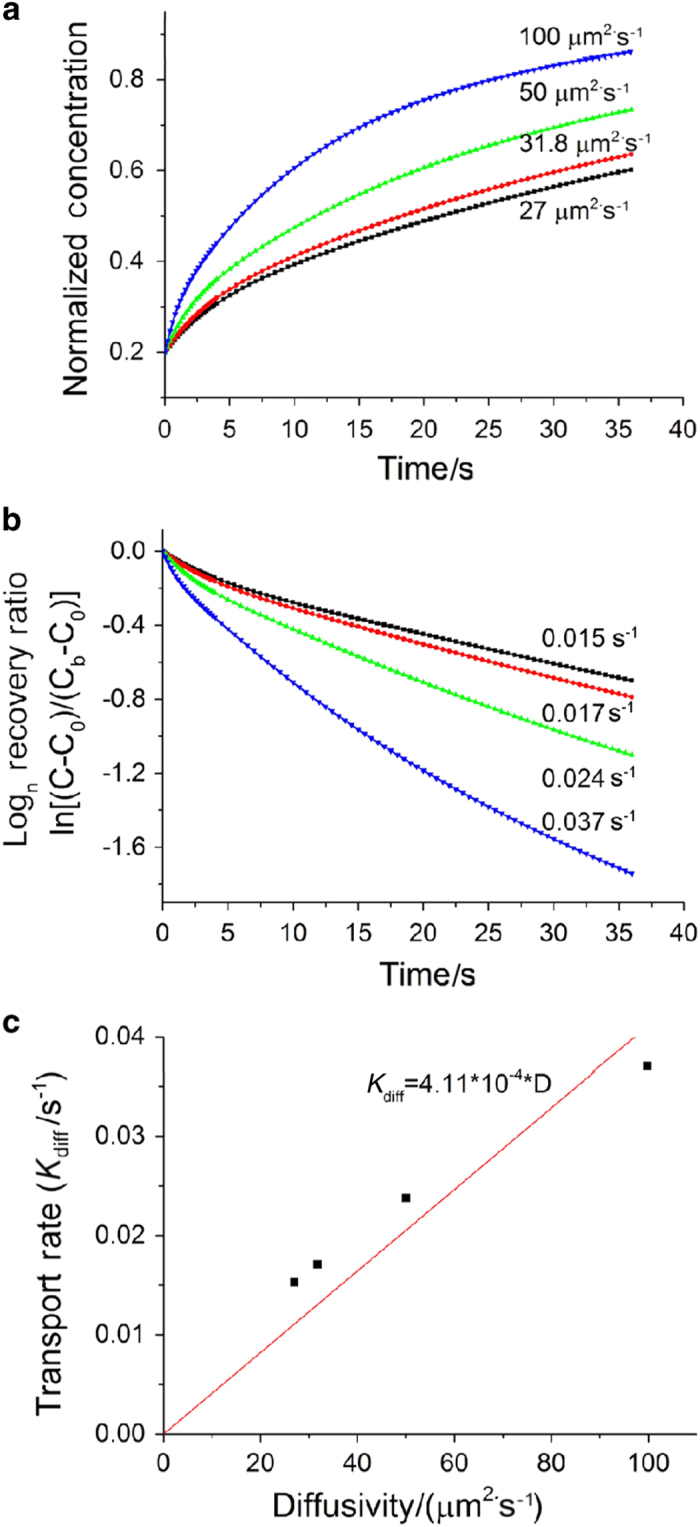
The tracer concentration (**a**) and the logarithm of the recovery rate (**b**) at the FRAP site under non-loaded condition using the segment biphasic model. (**c**) The model correctly predicted the nearly linear relationship between the transport rate and diffusivity in agreement with theoretical predictions (Wang *et al.* ^16^).

For the non-loaded condition, the solute concentration at the FRAP site was shown to increase with time and the rate of increase varied with diffusivity (Figure 4a). The transport rate, *K*, shown as the slope of ln[(*C−C*_0_)/(*C*_b_ *−C*_0_)] vs time curve (Figure 4b), was slightly higher initially and gradually reached a steady state (constant slope). This steady-state transport rate (*K*_diff_) was nearly linearly proportional to the solute diffusivity (*K*_diff_=4.11×10^−4^×*D*, Figure 4c). In particular, a diffusivity of 31.8 μm^2^·s^−1^ in the 3D porous model was found to best match the experimentally observed *K*_diff_=0.017 s^−1^ of sodium fluorescein (376 Da).^27^

**Figure 5:**
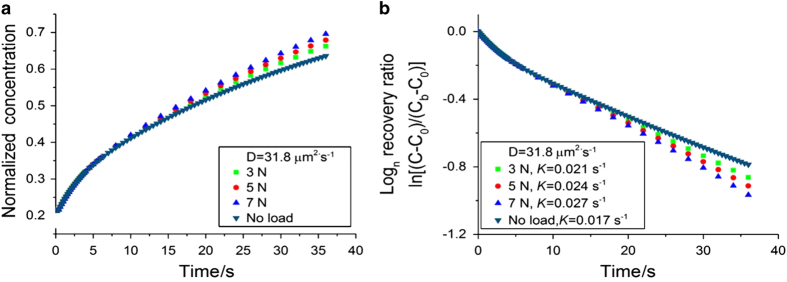
The tracer concentration (**a**) and the logarithm of the recovery rate (**b**) of sodium fluorescein under loaded and non-loaded conditions. A transport enhancement of 1.24 was found for 3 N loading, which is comparable with previous experimental measurements. As anticipated, higher loads (5 N and 7 N) result in greater transport enhancements.

This tissue diffusivity of 31.8 μm^2^·s^−1^ was then used to simulate the loaded condition (Figure 5). From the time courses of solute concentration (Figure 5a) and the logarithm of the recovery at the FRAP site under loaded and non-loaded conditions (Figure 5b), the transport enhancement (*K*_load_/*K*_diff_) was 1.24 for the 3N loading 3N, which fell within 1 s.d. above or below from the previously obtained experimental mean value (*K*_load_/*K*_diff_=1.31±0.24).^27^ In addition, we ran FRAP simulations with higher loads (5 N and 7 N). As anticipated, we observed faster fluorescence recovery and greater transport enhancement as load magnitude increased (Figure 5). Taken together, these results provide strong evidence that supports the use of FEBio segment transport model to predict the pore pressure and fluid flux in mechanically loaded bone.

### Results from the segment transport model

If not stated otherwise, the results presented in this section were obtained using the following parameters: a peak force of 3 N, material properties of 20 GPa Young’s modulus, 0.33 Poisson’s ratio, 15.4% porosity, and permeability of 2.8×10^−20^ m^2^.

#### Pore fluid pressure field

**Figure 6:**
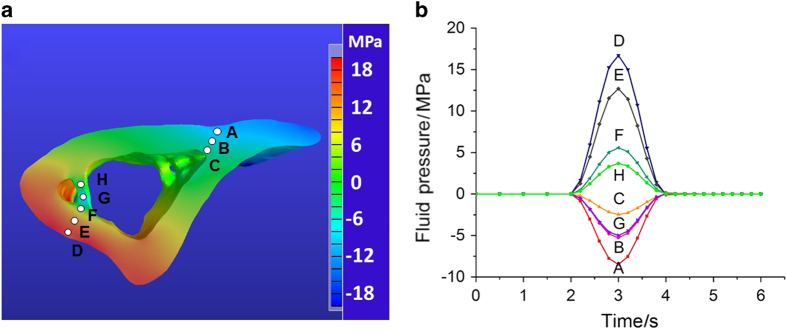
FEBio simulation of fluid pore pressure in the segment model. (**a**) The pore fluid pressure field at *t*=3 s at the cross-section containing the FRAP site (location A). (**b**) The temporal fluid pressure changes at the cortical (A–F) and trabecular sites (G, H) during one loading cycle (Figure 1b).

The pore fluid pressure distribution (Figure 6) was obtained at *t*=3 s when the loading peaked at 3 N (Figure 1b). In general, negative pressures were found in the regions under tension and positive pressures in the compressed regions, and the pressure magnitude in the cortex increases with the distance to the neutral plane (Figure 6a). Negative pressures were found in the trabecular area adjacent the cortex under compression (location G shown in blue shades), suggesting a more complex local loading pattern there. The temporal changes of the fluid pressure were shown during one loading cycle in Figure 6b, where location A (corresponding to the FRAP site) experienced a peak pressure of −8 MPa, and a high 17 MPa pressure was found in location D. Pressures in the trabecular locations (G and H) were relatively smaller in magnitude. Reducing permeability by one or two orders of magnitude (2.8×10^−21^ and 2.8×10^−22^ m^2^), peak pressure was found to vary slightly (<10%) or modestly (<25%) at locations A (+7% and +15%), B (+6% and +15%), C (−9% and −17%), D (+7% and +18%), E (+4% and +14%), F (−6% and −17%), G (+8% and +24%), and H (−6% and −18%), respectively.

#### Fluid flow field

**Figure 7:**
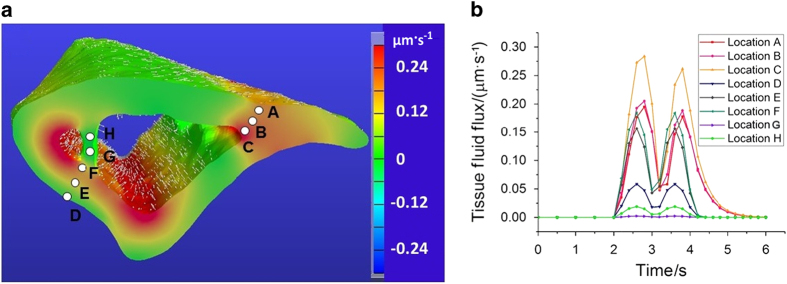
Load-induced fluid flux at tissue level. (**a**) The distribution of the fluid flux magnitude and the flow direction at *t*=2.6 second during the loading phase. (**b**) The temporal changes of the fluid flux at several selected locations in both cortical (locations A–F) and trabecular sites (locations G–H).

The local distribution of load-induced fluid flux could be obtained from the segment model. The local fluid flow varied cyclically as a function of time and a snapshot of the fluid flow field at *t*=2.6 s is shown in Figure 7a. The flows for the surface elements are shown with the vectors, with the length indicating flow magnitude and arrow indicating the flow direction; and the flow magnitude for other elements are indicated with pseudo-colors (Figure 7a). Overall, higher flow rates were found near the endosteal surfaces. The temporal profiles of flow magnitude at the selected locations are shown in Figure 7b. Comparing with fluid pressure that dropped to zero after *t*=4 s in most locations (Figure 6b), fluid flow at location B and C persisted till *t*=5 s (Figure 7b). Among all those selected locations, location C near the endosteal surface experienced the largest flux. Fluid flow was also found in the trabecular site (as shown in locations G and H), although the flux was relatively smaller than that in the adjacent endosteal cortical bone (Figure 7).

### Results from the ultrastructural canalicular flow model

Scaling based on the LCS porosity (15.4%), the peak canalicular flux was predicted to be 6.5-fold higher than that at the tissue level, varying from 0.02 to 1.84 μm·s^−1^ (Table 1) for cortical sites (A–F) and trabecular sites (G and H) (Figure 6). Site-dependent variations were also found for the shear stress acting on the osteocyte cell process and the two fluid-related stimulating forces such as fluid shearing force and drag force (Table 1). Consistently, the fluid drag force was approximately one order greater than the shear force.

## Discussion

The anabolic effects of mechanical forces have long been appreciated by the musculoskeletal research community.^1,4^ Clinicians routinely recommend exercise and physical activity to patients when treating and managing osteoporosis, osteoarthritis, and other medical conditions (www.cdc.gov). Quantifying the mechanical stimulation, however, remains a challenge, because of the complex structure and inhomogeneous material properties of the bone tissue^32^ and the different mechanical loading parameters associated with various exercise regimens.^9,10^ With the advances of imaging techniques, we are now able to reconstruct the 3D structures of bones with sufficient details from the whole-bone level (on the order of 0.1–1 m), individual osteon/trabecula level (on the order of 0.2 mm), and down to the cellular level (on the order of 0.01 mm).^39^ One striking feature of the bone tissue is the multiscaled, interconnected pore system housing the cellular components in bone, spanning the central marrow cavity, vascular channels, and the LCS.^9^ These pores are saturated with interstitial fluid, providing a continuous pathway for nutrient supply, waste removal, and exchange of signaling molecules. Furthermore, when bone is subjected to mechanical strains during exercise, it behaves as a stiff sponge: pore pressure builds up and fluid is forced to flow within the bone matrix through the pores.^9^ The osteocytes residing in the bone thus experience matrix deformation, fluid pore pressure, fluid shear force, as well as fluid drag force due to the pericellular tethering fibers.^9^ These mechanical parameters have been quantified using various engineering techniques. Matrix deformations have been measured with strain gages and imaging correlation methods.^3,5^ FRAP-based imaging techniques in combination with mathematical models provide measures of fluid and solute flows at the canalicular level under loading,^27^ allowing us to predict the various forces (shear and drag) that can trigger cellular responses.^28,29^ These data greatly enhance our understanding of the mechanosensing and mechanotransduction processes in bone that underlie the anabolic power of physical activities. However, these measures are currently limited to regions close to bone surfaces. The objective of this study was to expand the mapping of mechanical stimulations to the inner portions of the bone in a multiscale manner.

This study demonstrated the feasibility of performing such comprehensive mapping using an image-based FEA platform. This approach is consisted of three coupled models at the whole bone, bone segment, and single canaliculus levels. Comparing with previous models,^19–26^ the current model is unique by incorporating the ultrastructural model to predict loading effects (in terms of fluid shear and drag forces) that are highly relevant to the functioning of osteocytes. The model is physically sound, where the segment model is coupled with the whole-bone level through load-induced displacement fields, and the intrinsic LCS pores couple the segment model with the single canaliculus model. We also took advantage the available experimental data (on cortical bone) to extensively validate the model. The optically measured strain data^27,35^ were used to compare with the model outputs, ensuring that the material properties and the deformation coupling scheme were appropriate. The FRAP diffusion and transport enhancement measures^16,27,34^ were used to compare with the biphasic model in FEBio, enhancing our confidence of the choices of model parameters and boundary conditions. Using the validated models, we showed that loading-induced cellular stimulations such as pore pressure, fluid flow-induced shear stress and/or drag force acting on osteocytes could be mapped at both cortical and trabecular sites (Figures 6 and 7; Table 1). These quantitative data would help us to better understand which loading-induced parameters, including but not limited to matrix deformation, pore pressure, fluid shear, and fluid drag, correlate well with the spatial distribution of *in vivo* bone formation.

As with any model, our model has its limitations and assumptions. (1) The FE model was generated from a single biological sample, limiting the adoption of statistical analysis. As the goal of this study was to demonstrate the feasibility of modeling bone fluid flow, this simplified approach was chosen to remove any potential variabilities from the bone geometry and thus allow the study to better focus on validating model parameters and coupling schemes. With the procedure being streamlined and computational power ever increasing, the methodologies described herein can be applied for multiple samples to account for variations on bone anatomy. (2) The scaling factor between tissue-level fluid flow and the canalicular-level fluid flux was assumed to depend on the LCS porosity (15.4%), which was measured using confocal imaging. As noted earlier, this value may be an overestimation due to the scattering of fluorescence signals and axial stretching in the point spread function.^41^ Indeed, smaller porosity values (1%–5%) have been reported using methods based on electron microscopy and x-ray computation topography.^39^ Were the LCS assumed to be 1.5%, the model outputs (Table 1) would be expected to be nearly one order of magnitude higher. (3) The permeability we used in the model (2.8×10^−20^ m^2^) was based on our experimental measurement in dog bone.^38^ Large variations (in several orders of magnitude) of permeability have been reported in the literature.^39^ We also tested the sensitivity of the model outputs to permeability. As the permeability was reduced for one or two orders of magnitude (2.8×10^−21^ and 2.8×10^−22^ m^2^), fluid flow velocity was found to decrease compared with the values presented here (that is, 22% and 0.25% for locations A–C, and 69% and 15% for locations D–F, respectively). We thus conclude that accurate permeability measurement is the key to predict fluid flow velocity in the model. (4) We assumed sealed boundaries in our segment model for faster convergence in solute concentration simulations. This idealized condition was not fully compatible with *in vivo* situation where periosteum was found to be permeable for fluid and small solute.^44,45^ Leaky permeability should be considered for future modeling. (5) We assumed a biphasic material with isotropic linear elastic solid phase, which has constant isotropic permeability and constant isotropic solute diffusion coefficients. Previous studies^33^ indicated that the anisotropy of bone has an important role in the occurrence and distribution of the fluid flow in bone, which should be quantified in future experimental and modeling studies. Despite these limitations, our model was demonstrated to serve as a promising platform that would allow in-depth studies of local loading environment, which may help identify the important mechanical factors that drive bone’s response to loading and disuse in normal and disease conditions.

## Figures and Tables

**Table 1:** Cellular stimulation forces

Locations	Canalicular flow velocity/(μm·s^−1^)	Shear stress/Pa	Shear force/(pN·μm^−1^)	Drag force/(pN·μm^−1^)
A	1.27	0.17	0.08	0.75
B	1.33	0.18	0.09	0.78
C	1.84	0.25	0.12	1.08
D	0.38	0.05	0.02	0.22
E	1.02	0.14	0.07	0.6
F	1.2	0.16	0.08	0.7
G	0.02	0.003	0.001	0.01
H	0.12	0.02	0.008	0.07
